# A Systematic Literature Review of Consumers' Cognitive-Affective Needs in Product Design From 1999 to 2019

**DOI:** 10.3389/fnrgo.2020.617799

**Published:** 2021-02-03

**Authors:** David Ribeiro Tavares, Osiris Canciglieri Junior, Lia Buarque de Macedo Guimarães, Marcelo Rudek

**Affiliations:** ^1^Industrial and Systems Engineering Graduate Program (PPGEPS), Polytechnic School at Pontifical Catholic University of Paraná, Curitiba, Brazil; ^2^Production Engineering Graduate Program (PPGEP), Federal University of Rio Grande do Sul, Porto Alegre, Brazil

**Keywords:** cognitive, affective, consumer, product design, systematic review, state of the art

## Abstract

Understanding consumer cognitive and affective needs is a complex and tricky challenge for consumer studies. Creating and defining product attributes that meet the consumers' personal wishes and needs in different contexts is a challenge that demands new perspectives because there are mismatches between the objective of companies and the consumer's objective, which indicates the need for products to become increasingly consumer-oriented. Product design approaches aim to bring the product and consumer closer together. The objective of this study is to investigate the application of the cognitive and affective needs of the consumer in product design through a systematic review of the literature of publications carried out in the last 20 years. This article selects research carried out in the specific area of cognitive and affective product design and defines the state of the art of the main areas, challenges, and trends. The conclusion that was reached is that cognitive approaches have been updated, are more associated with technology, and so are focused and oriented toward the ease and friendliness of the product. In contrast, affective approaches are older and focus on the quality of life, satisfaction, pleasure, and friendliness of the product. This review indicates that the emotional focus of change for cognitive complexity is due to an understanding of the affective and emotional subjectivity of the consumers and how they can translate these requirements into product attributes. These approaches seem to lose their strength or preference in the areas of design and engineering for more rational and logical cognitive applications, and therefore are more statistically verifiable. Advances in neuroscience are focused on applications in marketing and consumer psychology and some cognitive and affective product designs.

## Introduction

Cognitive and affective product design is strategic for companies who wish to create deep connections with consumers through meaningful associations (Orth and Thurgood, 2018). These connections are valued for having intrinsic links with their beliefs, experiences, memories, people, places, or even personal values (Noble and Kumar, 2008). Thus, the Product Design (PD) and New Product Development (NPD) teams seek to understand which main cognitive and affective elements exist in the subjective product experience, relevant to consumer purchase intention and choice (Homburg et al., 2015).

The fact is that some products can be both comfortable and pleasant to use and consume, and thus promote both functional and “cognitive” as well as hedonic and “affective” experiences (Crilly et al., 2004; Khalid and Helander, 2004, 2006; Khalid, 2006; Seva and Helander, 2009; Wrigley, 2013). In previous reviews, these authors emphasize that such characteristics lead consumers to achieve their personal goals through functional, aesthetic, symbolic, semantic, formal, appearance, and status products, among many others. The design of the product aims to conceive and develop products that meet the needs and preferences of the consumer whether by better usability or functionality (Li and Gunal, 2012; Greggianin et al., 2018). They create not only a product more pleasant and accessible to use and consume but also products that accommodate for style and aesthetic beauty, hedonic pleasure, sympathy, and other interests (González-Sánchez and Gil-Iranzo, 2013). Through the evaluation and translation of opinions, the engineers and designers seek, to some extent, to produce happiness in the consumers' mind (Demirbilek and Sener, 2003). However, the opinions are individual and subjective, resulting from the use or consumption experience, or product experience (Schifferstein and Spence, 2008).

There were significant advances in product design before 1999, considering the processes of evaluation and the translation of consumers' cognitive and affective aspects. Among the relevant approaches found, Frijda (1986) deepened the research on emotions in products, focusing initially on facial expressions. For Frijda, emotions would tend to engage in behaviors influenced by the person's needs. Norman (1988) sought to include consumer accessibility in product design through resources with intense affective and emotional impact, popularizing the term user-centered design and simplifying the product's usability through greater functionality. Hauser and Clausing (1988) addressed quality as an essential requirement to meet consumer needs. The basis of the quality house was created so that product design activities could be carried out based on the wishes and needs of consumers. Another featured application was the kansei engineering methodology, as according to Nagamachi (1989), this methodology aims to implement the feelings and demands of consumers in the operation and design of the product. This author proposed a methodology to measure psychological aspects, understood as the consumer's kansei.

In the field of product design, Desmet (2003), Norman (1988), Jordan (1998), and Green and Jordan (1999) were pioneers in delving deeper into the product's affective and cognitive characteristics and in associating this information with the consumer's different cognitive and emotional levels. Since then, different research fields have studied ways of meeting consumers' subjective needs and preferences at different psychological levels (Hong et al., 2008). The objective is to attract the consumer with products that provide innovative experiences with intense cognitive and affective impacts (Kumar Ranganathan et al., 2013).

Ellsworth and Scherer (2003) highlight that, while affection refers to sentimental responses, cognition is used to interpret, comprehend, and understand the experience. Cognition understands and comprehends what is perceived, while affection promotes the learning and experience feeling in the interaction with the product. Norman (2004) argues that the cognitive system gives meaning to the world while the affective one is critical to it. Both complement each other and each system influences the other, with cognition providing affection and being affected by it (Ashby et al., 1999; Coates, 2003; Crilly et al., 2004). However, the strategy of many designers is not clear on the importance of associating cognitive and affective needs of the consumer with the cognitive and affective attributes of the product, which creates a problem for the research field in product design (Crilly et al., 2004; Khalid and Helander, 2004; Kumar Ranganathan et al., 2013; Zhou et al., 2013; Gómez-Corona et al., 2017; Hsu, 2017; Jiao et al., 2017). Khalid and Helander (2006) state that the consumer perceives reality in an affective (intuitive and experiential) and cognitive (analytical and rational) way, and separating emotion from cognition is a major deficiency of psychology and cognitive science in general. Emotions are not the cause of rational thinking, but they can motivate an interest in objectivity. Rational thinking affects feelings and affective thinking influences cognition. Therefore, the phenomena are inseparable.

Nevertheless, few integrated applications of cognitive and affective needs in product design are found in the literature. Although the opinion among researchers is that the cognitive and affective human systems belong to a single source of informational processing, the understanding and evaluation of the functioning of these systems are considered essentially “closed,” a “minefield” (Khalid, 2006; Khalid and Helander, 2006), or a real “black box” (Zhou et al., 2013; Diego-Mas and Alcaide-Marzal, 2016; Jiao et al., 2017). Although there have been significant advances in the understanding of the combination of cognitive and affective systems (Damasio, 2001; Damasio and Adolphs, 2001), areas of engineering and product design still face difficulties in uniting the two mental processes in the same applications. The justification for this research is to investigate the importance of advancing the study of consumers' cognitive and affective needs in the manner of product characteristics and attributes which is considered an essential path for product design (Kumar Ranganathan et al., 2013).

In this sense, this article seeks to select the research carried out in the specific field of cognitive and affective product design and to identify the main areas, challenges, and trends of the applications as well as to advance the investigation of the problems which justify this research. From this, what would be the main research carried out in the last 20 years on the application of cognitive and affective needs regarding the characteristics and attributes of product design that can contribute to the advancement of consumer research?

## Methods and Materials

### Systematic Literature Review (SLR)

**Figure 1:**
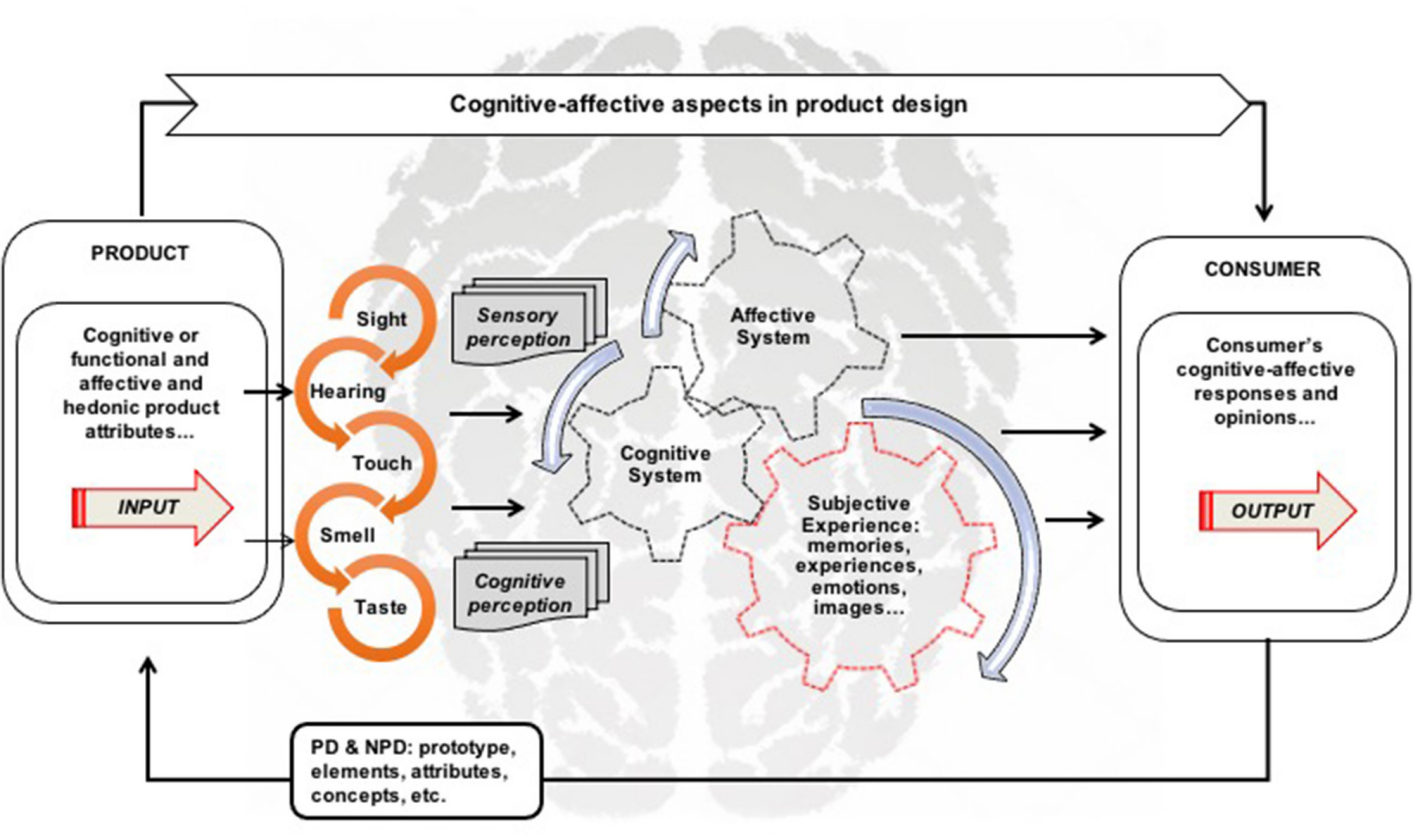
Conceptual framework of the cognitive and affective aspects in product design.

Through the studies presented so far, Figure 1 shows the starting point for the beginning of the research. This focuses on the cognitive and affective aspects derived from the product and the consumer. On the consumer side it involves senses of sensory perception, cognitive, and affective mental systems, and subjectivity experience when interacting with the product. On the product side, it generally involves cognitive attributes (functionality, usability, etc.) and affective attributes (pleasure, hedonism, pleasantness, etc.). This information is usually captured, evaluated, translated, and applied to product design.

The practical applications of cognitive and affective aspects in the product design are summarized in the conceptual framework. To identify the most relevant literature related to the topics covered, this study conducted a systematic literature review (SLR) based on data from Cambridge Journals Online, Emerald Insight, IEEE Xplore, Scopus Science, Springer Link, Taylor and Francis, and other databases such as Google Scholar.

The SLR procedure is a research method that achieves results through information already described and published, which minimizes distortions and errors (Jesson and Lacey, 2006; Mattioda et al., 2015; Randhawa et al., 2016). The study selected only articles that were: (i) peer-reviewed; (ii) written in the English language; and (iii) published in the last 20 years (from 1999 to 2019). The 20-year period aims to meet analysis robustness and the synthesis of the topics covered by considering the largest possible number of approaches that define the research object.

The search keywords are derived from the framework presented, and the selection of the articles was defined based on the following terms: cognitive, affective, or emotional aspects, and product and new products design. Based on these terms, the study searched the following keywords in the databases based on the crossing of the two groups of words: (i) cognitive aspects (“cognition” or “cognitive,” “cognitive design”) and affective aspects (“affect” or “affective,” “affective design,” “emotion,” or “emotional” and “emotion/emotional design”); and (ii) product design: “product design” (PD), “product development process” (PDP), “new product development” (NPD).

#### The PRISMA Flow Diagram

**Figure 2:**
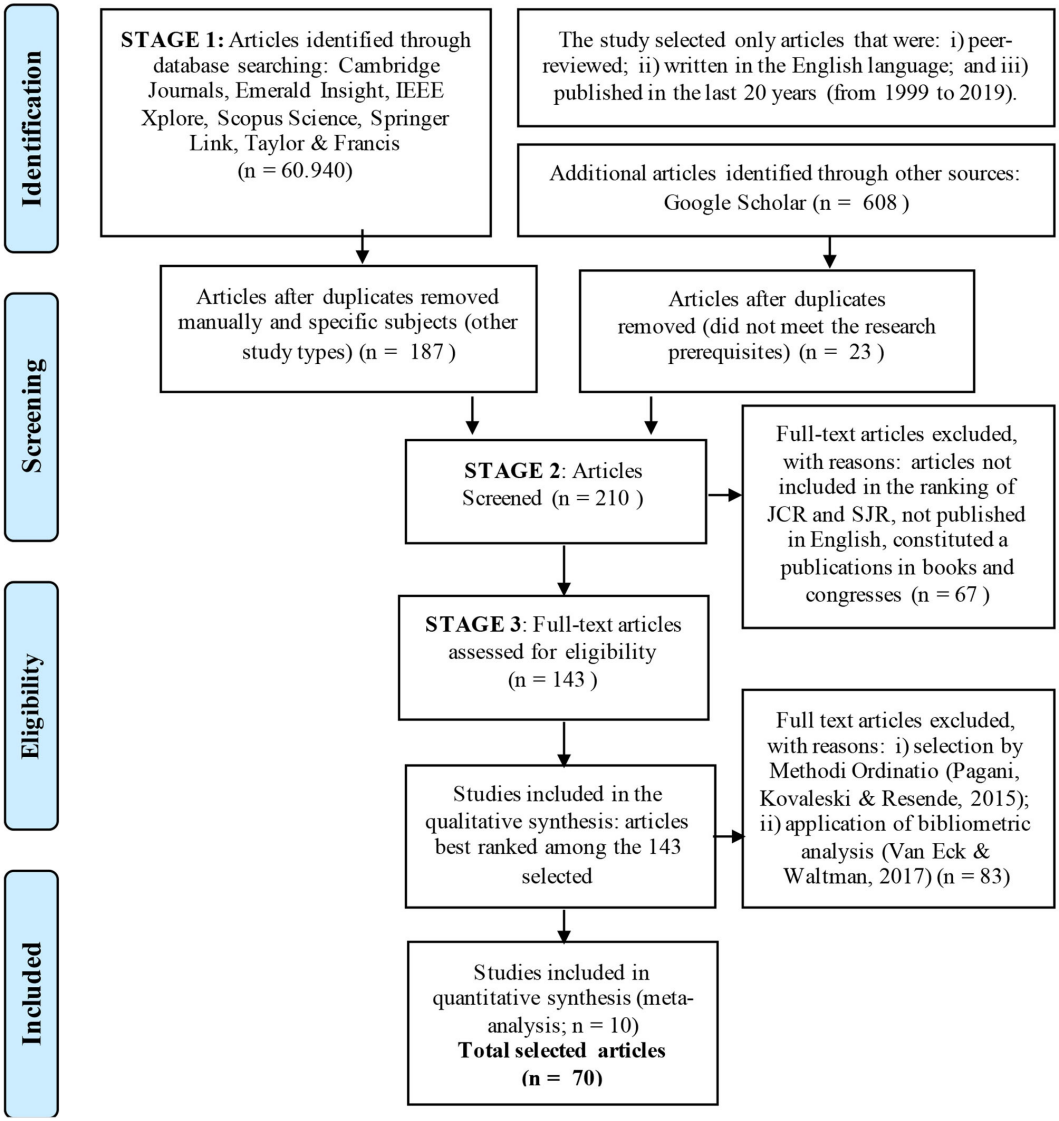
Flow diagram of systematic review process (based on the generic diagram in Moher et al., 2009).

The PRISMA flow diagram (Moher et al., 2009) was used to organize the SLR (Figure 2).

In the first *stage*, the research was based on the crosschecking of the keywords. The search result for any subject in the databases included 60,940 articles. After directing the research to only specific subjects considering only the keywords, the result included 187 articles. The research made among Google Scholar's open and available databases resulted in 608 articles.

After *identification*, in the second *stage*, the research pre-selected the articles. From the 187 articles, among those that contained in their keywords the terms defined in the preliminary research, 47 of them were excluded because they were duplicated in the sample. After the exclusion of duplicate articles, in a language other than English, and from publications in books and congresses, only 23 articles met the research prerequisites from the 608 found in the open database of Google Scholar. Another exclusion criterion was the removal of articles published in journals not included in the ranking of JCR (Journal Citation Ranking) and SJR (Scimago Journal Ranking) impact factor, a requirement considered important for the next SLR stage. The result was a gross portfolio of 143 base articles for the selection by relevance.

After *screening*, for the *third stage* for the *eligibility* of articles, a *qualitative synthesis* was initiated.

#### Qualitative Synthesis

The selection criterion was defined by applying the *Methodi Ordinatio* (Pagani et al., 2015) that uses the InOrdinatio index, the result of an equation that considers the “impact factor” relevance of the journal where the article is published, the “number of citations” and the importance of more “recent” works that have not yet obtained many citations from peers. In summary, the equation consists of adding the journal's impact factor, the number of citations the article received by its peers to a factor that considers the relevance of how recent the article is when considering its publication year, according to Equation (1):


(1) $$\begin{array}{l} InOrdinatio\text{ = (}IF\text{/1,000) + }alpha\\ {}^{*}[10-(ResearchYear-PublishYear)]+Citations \end{array}$$

where: (i) “IF” is the impact factor of the publication, (ii) “α” is a weighting factor that varies from 1 to 10, normally assigned by the researcher; (iii) “ResearchYear” is the year in which the research was developed; (iv) “PublishYear” is the year in which the article was published; and (v) “Σ Ci” is the number of times the article has been cited.

To identify the number of citations by peers, this study considered Google Scholar. The reason for this is the fact that several articles were not included in the main scientific databases that conduct bibliometric analyzes, and that calculate the number of citations by peers, such as Scopus, Proquest, or Elsevier. These databases did not show all articles selected in the initial search. Google Scholar presented all selected items in the gross portfolio after verification.

The “α” criterion was defined by the following formulation that takes into account the current publication status: “10” for publications made in the last 4 years; “8” for publications in the last 5–8 years; “6” for publications in the last 9–12 years; “4” for publications in the last 13–16 years; “2” for publications in the last 17–20 years; and “0” if there were any classic and relevant articles published more than 20 years ago and later inserted in the sample.

After the application of Equation 1 and data handling, the study obtained the InOrdinatio index of each article, for classification according to its scientific relevance for the research. The higher the value of the InOrdinatio index, the more relevant the article was considered. However, articles with more citations stood out in relation to the others and could leave some important studies out of the content analysis.

To solve this deficiency, the study developed a new criterion using the Ordinatio Method and applied it to reinforce the search for the most relevant articles for the research. The new criterion was configured through bibliometric analysis. The objective was to highlight the analysis through the articles initially selected by the research, considering the impact factor of the publication, the number of citations by the peers, and as a complementary addition verify the strength of the keywords chosen for the SLR, both in the occurrences of citation and in the total strength of the correlation links with other works in the gross portfolio.

#### Quantitative Synthesis

To improve the *eligibility* of the chosen papers the study considered and calculated all terms available in the title and keywords of the 143 articles in the gross portfolio. The objective was to compensate for the difference in the volume of citations by peers found in the oldest articles compared to the most recent and, therefore, little cited. To achieve this, the study developed a new adherence factor in order to verify the importance of articles that were not included in the previous selection. It also considered the article's proximity to the main topics covered, as presented at the beginning of this review, which justified further research.

The *software Vosviewer 1.6.11*, designed for bibliometric network analysis (Van Eck and Waltman, 2017), was used to identify the keywords with the highest occurrence and full strength of links among the main terms addressed by peers from the 143 articles in the gross portfolio. In the software application, the examples were obtained as a result of bibliographic coupling links among publications, co-authoring links among researchers, and occurrence links among terms or keywords. Among the options for a search item, there were links between different terms that point to the number of links between keywords. The total strength of the links between the keywords showed more than one link and the co-occurrence between the terms, which pointed to the number of publications in which the terms occurred together. The higher the numerical value displayed, the stronger the link or the strength of the link between the terms or keywords.

The articles containing the highlighted keywords (considered here with only four or more occurrences—Table 1) received the sum of the occurrences volume and the total strength of the links for each keyword. Subsequently, the sum of the volumes of each keyword was added to the value of their InOrdinatio, as shown in Equation (2):


(2) $$\begin{array}{l} Final\;Selection\;=\;InOrdinatio\;+\;\sum No\;of\;occurrences\\ +\;\sum \;Total\;link\;strength \end{array}$$

With the application of Equation 2 as a determinant for the selection of articles, articles not considered in the initial qualitative verification (Equation 1) were included in the sample.

**Table 1:** Terms or keywords with an occurrence equal to or greater than four.

**Keywords**	**Occurrences**	**Total strength of links**
Product design (PD)	57	242
Affective design (AD)	16	68
Kansei engineering (KE)	14	62
New product development (NPD)	14	64
Emotional design (ED)	11	56
Cognition	9	70
Affective product design (APD)	7	20
Aesthetics	7	34
User experience (UX)	5	32
Emotion (s)	7	33
Affective response (s) (AR)	5	25
Usability	4	35
Learning	4	20
Inclusive design (ID)	4	17
Perception (s)	4	31

## Results

Table 2 shows the result of the SLR (70 articles). These articles compose the sample for the analysis and discussion of the results. It presents the main authors and topics covered highlighted in the research field. It is possible to verify the results of the qualitative synthesis (Equation 1) and the quantitative synthesis (Equation 2) in detail. The volume of citations and the impact factor of each paper, the year outlining the topicality of the subject, as well as the number of occurrences and strength of the links between the titles and the keywords of the research. The methodology used can be easily replicated in future research.

**Table 2:** Classification of the final selection by relevance and impact in the research.

***N***.	**Author (s)**	**Equation 2**	**Equation 1**	**(InOrdinatio)**	**Citation**	**Impact factor**	**Publish year**	PD (57 + 242)*	**AD (16 + 68)**	**KE (14 + 62)**	**NPD (14 + 64)**	**ED (11 + 56)**	**Cognition (9 + 70)**	**APD (7 + 20)**	**Aesthetics (7 + 34)**	**UEX (5 + 32)**	**Emotion (s) (7 + 33)**	**AR (5 + 25)**	**Usability (4 + 35)**	**Learning (4 + 20)**	**ID (4 + 17)**	**Perception (s) (4 + 31)**
1	Jiang et al., 2015a	1,238	863		883	0.96	2004	X							X							X
2	Nagamachi, 2002	1,072	697		711	0.96	2002	X		X												
3	Demirbilek and Sener, 2003	688	282		306	0.99	2003	X				X					X					
4	Mehta and Zhu, 2009	682	603		603	13.25	2009						X									
5	Rindova and Petkova, 2007	680	346		358	6.55	2007	X														X
6	van Kleef et al., 2005	651	573		589	1.14	2005				X											
7	Page and Herr, 2002	610	227		241	2.98	2002	X	X													
8	Khalid and Helander, 2006	595	172		184	0.55	2006	X	X								X					
9	Jiao et al., 2017	583	84		4	1.39	2017	X	X				X			X						
10	Kumar and Garg, 2010	572	113		107	2.98	2010	X					X		X		X					
11	Camargo and Henson, 2011	562	36		16	0.2	2011	X	X	X		X										
12	Guo et al., 2016	555	72		2	0.37	2016	X		X		X			X							
13	Gilal et al., 2018	553	91		1	0.86	2018	X	X				X									
14	Khalid, 2006	550	167		185	0.96	2006	X	X													
15	Hsu et al., 2018	536	93		3	0.83	2018	X					X					X				X
16	Aftab and Rusli, 2017	523	81		1	0.8	2017	X		X		X										
17	Huang et al., 2012	520	78		54	0.55	2012	X		X		X										
18	Khalid and Helander, 2004	520	137		157	0.43	2004	X	X													
19	Wiecek et al., 2019	519	100		0	3.79	2019	X					X		X							
20	Zhou et al., 2013	518	103		71	1.39	2013		X				X			X						
21	Wang et al., 2018	511	98		8	0.65	2018	X	X									X				
22	Lewis and Neider, 2017	508	91		11	0.34	2017	X					X						X			
23	Greggianin et al., 2018	507	90		0	0.33	2018	X							X	X	X					
24	Huang et al., 2014	505	63		23	0.55	2014	X		X		X										
25	Blackler et al., 2010	502	124		118	0.96	2010	X					X									
26	Lin et al., 2012	502	43		19	0.55	2012	X	X	X												
27	Langdon et al., 2007	493	94		106	0.35	2007	X					X								X	
28	Hill and Bohil, 2016	490	73		3	0.34	2016	X					X						X			
29	Hsiao and Chen, 2006	489	160		178	0.55	2006	X										X				
30	Chen et al., 2016	487	74		4	0.22	2016	X					X									X
31	Yang and Shieh, 2010	484	79		73	1.33	2010	X		X								X				
32	Miesler, 2011	483	70		54	0.52	2011	X										X				X
33	Mieczakowski et al., 2013	480	44		12	0.35	2013	X					X			X					X	
34	Perttula and Sipilä, 2007	478	179		191	0.65	2007	X														
35	Liu and Tong, 2018	477	94		4	0.38	2018	X	X													
36	Lo and Chu, 2014	477	53		13	0.37	2014	X	X						X							
37	Hoegg et al., 2010	474	134		128	2.98	2010	X							X							
38	Murphy, 2015	474	61		1	0.34	2015	X					X									X
39	Zhai et al., 2009	471	88		88	0.55	2009	X	X													
40	Xu et al., 2012	469	27		3	0.45	2012	X		X		X										
41	Yang, 2011	466	91		75	1.33	2011	X		X												
42	Jiang et al., 2015b	464	81		33	1.59	2015	X	X													
43	Langdon et al., 2010	463	40		34	0.35	2010	X					X							X	X	
44	Zayas-Cabán and Chaney, 2014	457	40		0	0.34	2014	X					X						X			
45	Félix and Duarte, 2018	456	90		0	0.2	2018	X				X										
46	Karim et al., 2017	455	81		1	1	2017	X									X					X
47	Bahn et al., 2009	448	65		65	0.37	2009	X	X													
48	Landwehr et al., 2012	437	54		30	1.36	2012	X	X													
49	Landwehr et al., 2011	436	137		121	6.85	2011	X														
50	Artacho-Ramírez et al., 2008	434	56		62	0.55	2008	X					X									
51	Orth and Thurgood, 2018	429	90		0	0.52	2018	X									X					
52	Spendlove, 2008	428	28		34	0.56	2008	X								X	X			X		
53	Seva et al., 2007	427	101		113	0.96	2007	X						X								
54	Hong et al., 2008	427	44		50	0.55	2008	X	X													
55	Guastello et al., 2014	427	49		9	0.43	2014	X					X									
56	Li et al., 2014	426	48		8	0.39	2014	X					X									
57	Cheah et al., 2011	422	44		28	1.19	2011	X					X									
58	Van Rompay and Ludden, 2015	422	86		38	0.52	2015	X								X						
59	Nam and Kim, 2011	422	42		26	0.52	2011	X							X		X					
60	Yang and Chang, 2012	421	43		19	0.96	2012	X		X												
61	Noble and Kumar, 2008	418	79		85	1.3	2008	X									X					
62	Diego-Mas and Alcaide-Marzal, 2016	418	84		14	0.55	2016	X														X
63	Persad et al., 2007	411	91		103	0.35	2007	X													X	
64	Chen and Chu, 2012	409	43		19	1.19	2012	X				X										
65	Yang et al., 2016	406	72		2	0.27	2016	X														X
66	Seva and Helander, 2009	402	76		76	0.55	2009	X						X								
67	Cho et al., 2011	400	22		6	0.58	2011	X					X									
68	Fu Qiu et al., 2008	397	19		25	1.28	2008	X					X									
69	Seva et al., 2011	393	67		51	0.96	2011	X						X								
70	Wrigley, 2013	392	53		21	0.27	2013	X									X					

* *For example, PD (57 + 242) corresponds to the keyword product design, which contains 57 occurrences plus 242 as the total strength of the links between the other articles in the gross portfolio*.

The applications occurred in two large areas, as shown in Table 3. The detailed bibliometric analysis of the applications made it possible to organize the approaches in order of relevance: affective/emotional approach and cognitive approach.

**Table 3:** Occurrence of affective/emotional and cognitive product approaches.

**Affective/emotional approach**	**Nr. occurrences**	**Cognitive approach**	**Nr. occurrences**
Aesthetics	6	Cognitive engineering	3
Affective design	9	Cognitive ergonomics	3
Emotional design	6	Usability	3
Kansei engineering	8	Cognition	5
Affective product design	3	Inclusive design	4

### Cognitive and Affective Design Approach

**Figure 3:**
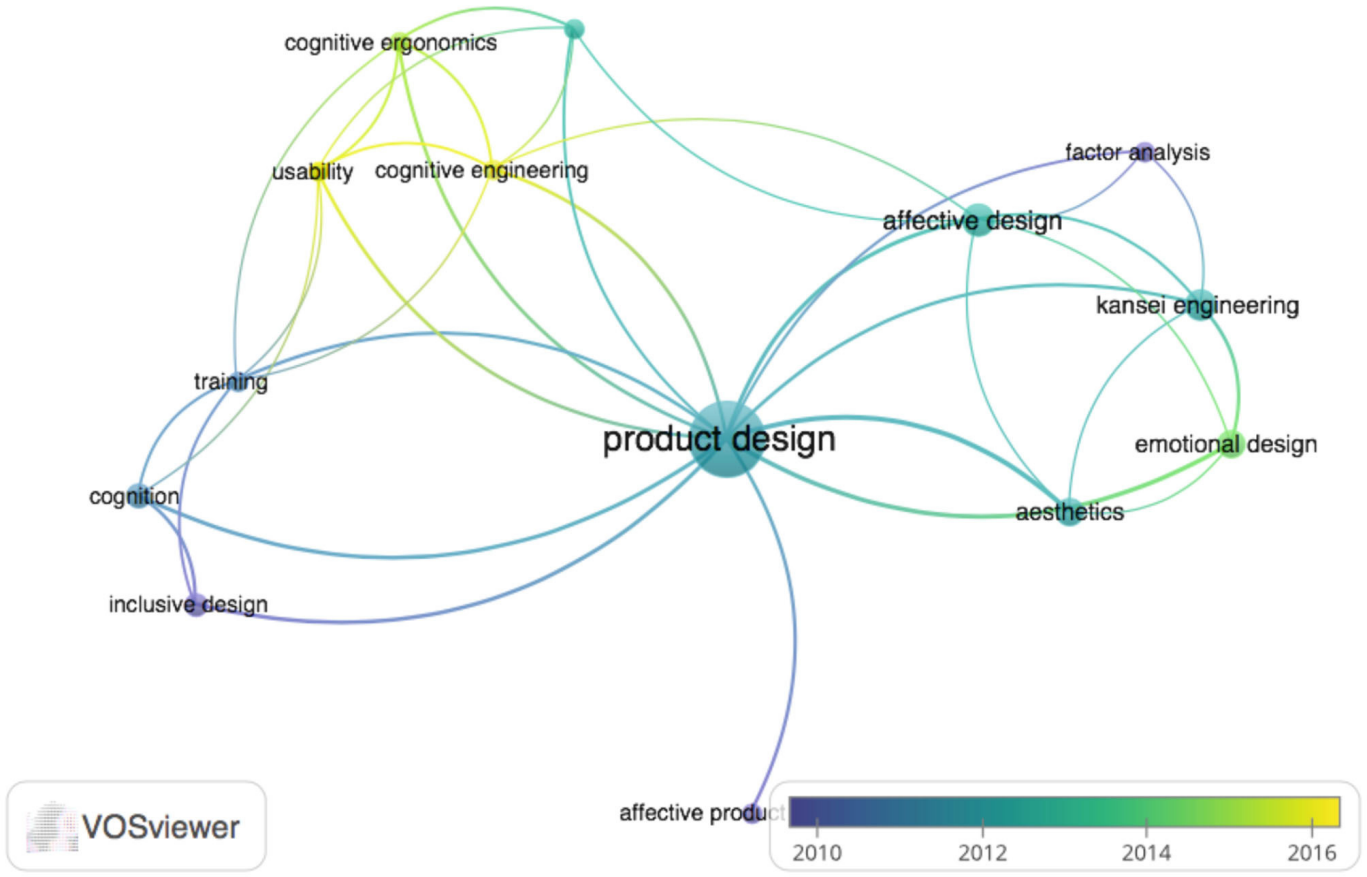
Network view of the application areas, with information from the publication year overlapped.

The networked view considers the overlapping data of information about the publication year and presents the timeliness of approaches. Figure 3 presents clusters of evident keywords in the articles. They are organized ranging from the “darkest” and oldest, to the “lightest” and most current, and show an important trend in the types of applications and topicality of the topics covered.

Applications in “usability” (Seva et al., 2011; Hill and Bohil, 2016), “cognitive ergonomics” (Chang and Chen, 2016; Montewka et al., 2017), and “cognitive engineering” (Li and Gunal, 2012) appear to be more current than applications in “affective design” (Jiao et al., 2006; Lu and Petiot, 2014; Jiang et al., 2015a), “kansei engineering” (Nagamachi, 2002; Xu et al., 2012; Mele and Campana, 2018), and “emotional design” (Guo et al., 2014). All cognitive and affective need applications are interconnected to the product design and indicate cognitive approaches more focused on product usability and functionality, while affective and emotional approaches are more focused on pleasure and consumption.

**Figure 4:**
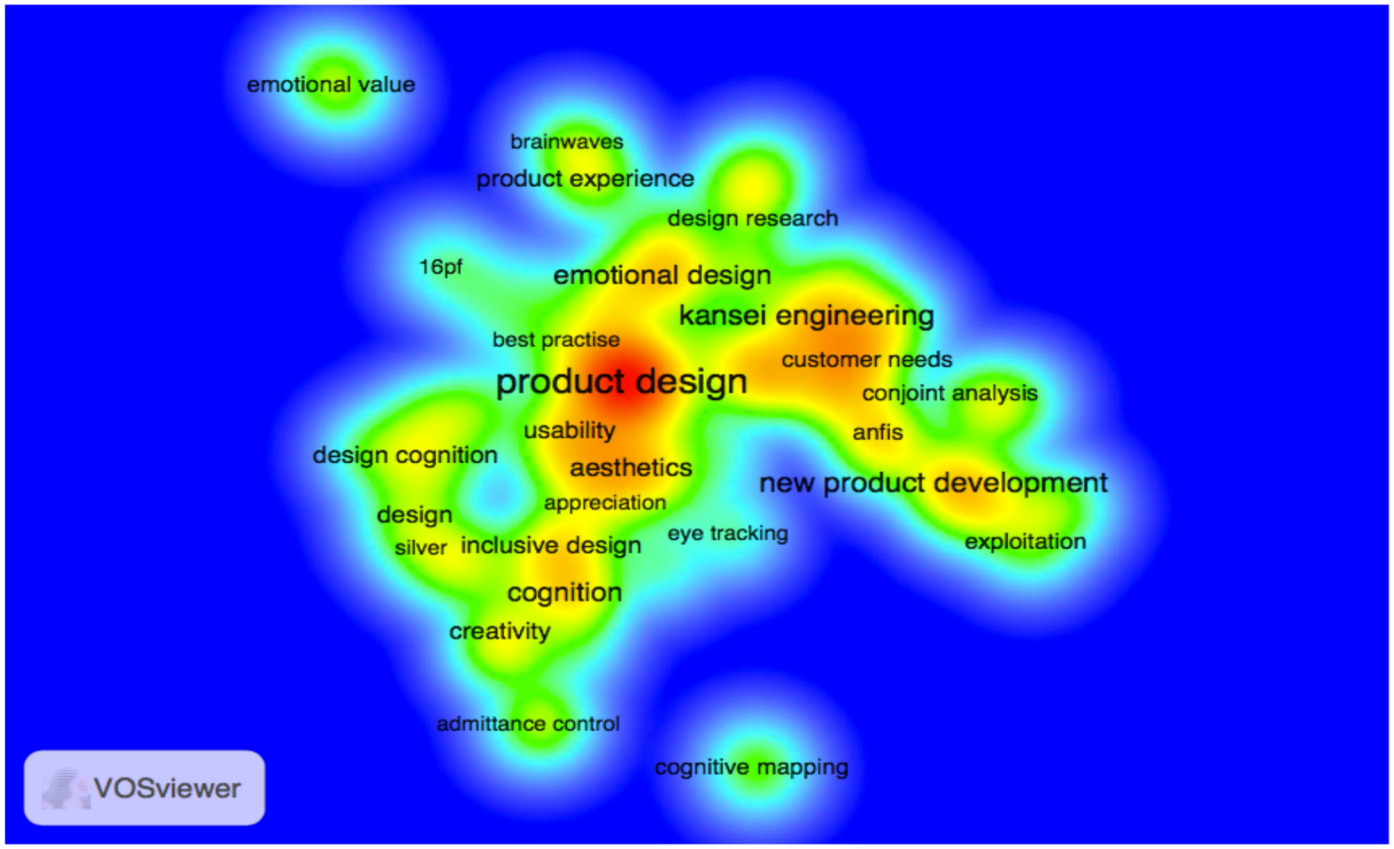
Visualization map of terms or keywords by density.

On one hand, there are approaches to ergonomics and cognitive engineering that direct them to usability and product quality (Seva et al., 2011), as well as learning and training aspects (Yang and Shieh, 2010; Hsu, 2017), or interaction design (Langdon et al., 2007; Faiola and Matei, 2010; Nam and Kim, 2011; Mieczakowski et al., 2013). On the other hand, there are approaches that seek to meet the consumer's most affective and emotional needs and preferences and, thereby, improve quality of life. These approaches focus on the affective design (Guo et al., 2016; Gilal et al., 2018) and emotional design (Félix and Duarte, 2018). The kansei engineering (KE) method is featured among the affective approaches and seeks to evaluate and translate the consumer subjective requirements into product attributes, as shown in Figure 4 in the density view of terms or keywords. The greater the occurrence of the terms, the greater the size of the letters and the more intense the colors presented (for example, warm, red). In addition, the closer a word is to the other, the greater the link strength between the terms, which shows the intensity of research in different types of approaches.

### Cognitive Design

Among the most current approaches (Figure 3), it is possible to mention the cognitive design application. Inclusive design (Langdon et al., 2007, 2010), education (Faiola and Matei, 2010; Lu, 2017; Kiernan et al., 2019), and learning and creativity approaches (Spendlove, 2008) are the most explored by researchers. They seek to evaluate and translate the product's usability and functionality attributes, making the interaction easier for the consumer, as for example when understanding the color effect (blue or red) on the performance of the user's cognitive tasks (Mehta and Zhu, 2009). According to Murphy (2015), there is an understanding that color should be used with a different code in the world of human-computer interactions, such as form or pattern fillings, in order to make the content accessible to everyone, including those with color vision deficits.

Some approaches aim to gather the perception of the consumer's image with the product form (Lin et al., 2012; Chen et al., 2016). Others aim to investigate the “noise” influences on visual cognitive responses to the design of human-oriented products (Cho et al., 2011).

There is strong evidence that a good design is important in the creation of products for intuitive use (Blackler et al., 2010). This makes it possible to assist in the inclusive interaction design, through a better understanding of the cognitive representations or through processes of producing mental images of designers and users (Mieczakowski et al., 2013). Inclusive design is relevant by differentiating the effects of easy-to-use consumer products from those difficult to use (Langdon et al., 2007). These data corroborate the growing demographic demand of an increasingly aging population, which should be included in product design (Lewis and Neider, 2017).

In many approaches, the cognitive application mixes with the affective application (Hsu et al., 2018), as there is still no clear or deeper explanation about the separation between the psychological functions and processes involved in the subjective experience of interaction between the consumer and the product (Khalid and Helander, 2004; Zhou et al., 2013). This problem is considered the true “black box” of content or substance knowledge that composes the internal and subjective processes of the functioning of cognitive and affective systems.

### Affective/Emotional Design

The approaches on affective/emotional product design are quite varied (Kumar Ranganathan et al., 2013). The affective and emotional satisfaction are objectives of most approaches on affective product design (Chan et al., 2018). These ones mix with emotional approaches and are synonymous in most applications. According to Chen and Chu (2012), consumers often make their purchasing decisions based on the product price, quality, and functionality. However, in many situations the perceived value influences the decision, which is always subjective and motivated by emotions. It is important to predict the perceived value of design alternatives based on the common language that target consumers and designers understand.

Other approaches seek to measure affective responses to consumer-oriented product design (Camargo and Henson, 2011). There are also approaches that measure the responses to the affective aspects applied to product design in order to improve the consumer's affective satisfaction (Hong et al., 2008; Zhai et al., 2009). Still others measure the reactions of the effects of product attributes on personal interactions, for which Lo and Chu (2014) propose a concept of socio-affective product design. The focus of affective approaches is always the consumer, their desires, personal interaction, quality of life, and satisfaction.

In relation to affective design, one of the most important tasks is to evoke specific affective responses through the manipulation of product form (Yang and Shieh, 2010; Yang, 2011; Diego-Mas and Alcaide-Marzal, 2016). The main objective of these approaches is to provoke positive affective and emotional responses in the consumer. Hsiao and Chen (2006) investigate the structure of the relationship between the product forms and consumer's affective responses. The product shape is increasingly important to provoke affective responses. By applying an evolutionary approach, Miesler (2011) examines affective responses in relation to facial features. When combining facial electromyography with assessments of a “baby's facial shapes” in order to assess innate emotional responses in the consumer, he discovered that, in this case, the participants presented more positive and affective responses. The results confirm that the resources acquired in an evolutionary manner affect the consumer's affective responses to the products' visual forms.

The emotional design and related approaches meet the vision of designers and manufacturers who understand consumption as the main objective of a product. They seek to generate and add value to the product through emotional design, trying to find a lasting connection between the product and consumer (Aftab and Rusli, 2017). The inclusion of aesthetic and functional attributes causes positive emotional experiences (Seva and Helander, 2009), which provide pleasantness and pleasure to the consumer, for example, in bra design (Greggianin et al., 2018).

Digital technology is also presented to apply to the consumer's emotional aspects in product engineering and design. In relation to the digital world, Nam and Kim (2011) seek to help designers to create meaningful products for the digital world while preserving the technology benefits. There is a great opportunity for design to increase the extra experiential value of products in a world with digital technologies. The approaches aim to add value to the product through important emotional attributes for the consumer. Sophisticated applications with smart neural networks and optimization methods are also used to meet emotional needs (Guo et al., 2016) and increase the consumer's quality of life (Félix and Duarte, 2018).

In summary, measuring and evaluating affective and emotional responses and projecting design elements or attributes (Camargo and Henson, 2011), attributes that provoke essentially positive affective and emotional reactions, are the focus of most approaches for a product's affective/emotional design.

## Analysis and Discussion

Different areas of product design seek to understand the relationship between product and consumer. Affective product design explores the most affective aspects between the product and consumer, as proposed by Khalid and Helander (2004), Khalid (2006), Khalid and Helander (2006), Seva and Helander (2009), Seva et al. (2011), and Diego-Mas and Alcaide-Marzal (2016). Cognitive-emotional product design proposes a more sentimental, visceral, and hedonic approach, as suggested by Crilly et al. (2004), Wrigley (2013), and Karim et al. (2017). Other approaches (e.g., Rindova and Petkova, 2007; Artacho-Ramírez et al., 2008; Li et al., 2014) mix innovation elements and cognitive and emotional aspects in the cognitive design. There is also the design approach of affective-cognitive experience product design with user's experience bias (e.g., Zhou et al., 2013; Jiao et al., 2017). These studies share common challenges, such as the complexity of understanding and evaluating the consumers' subjective cognitive and affective needs (Table 4), or understanding the interaction experience between the product and consumer, or even the product experience (Schifferstein and Hekker, 2011).

**Table 4:** Challenges in applications of consumer's cognitive and affective needs in product design.

**Areas**	**Sample, design, and measures**	**Challenges**
Affective product design	Artificial neural networks to model affective responses to the shape design of paddle tennis rackets and motorcycle helmets (Diego-Mas and Alcaide-Marzal, 2016). Systematic framework to conceptualize affective needs in the design of the hedonic and functional attributes of electronic devices in cars (Khalid and Helander, 2004). Review about crossing between cognitive and affective decision-making systems (Khalid, 2006). Measures of data on mood, effect of advance purchase, and purchase intention for aesthetic and functional attributes of cell phones (Seva and Helander, 2009). Measures of intense affection and perceived usability of attributes related to the functional and aesthetic shape of cell phones (Seva et al., 2011).	Understand heuristic-affective biases in the consumer's affective decision-making process; Integrate cognitive and affective systems in assessments; Understanding how products form attributes to evoke feelings that affect the consumer's intention and decision.
Cognitive-emotional product design	Review and framework about responses of cognitive and affective interaction in relation to visual domain of aesthetic, semantic, and symbolic aspects of the product (Crilly et al., 2004). Method about hedonic visceral rhetorical elements of the product has a significant role in determining responses such as consumer intention and decision (Wrigley, 2013). Consumer purchase intention through the measurement of facial expressions existing in digital watch photographs (Karim et al., 2017).	Demonstrating that cognitive and affective reactions and responses belong to the same process; Improve communication between design and consumer; Go beyond the functionality and usability attributes of the product; Understanding the bias of affective judgment and cognitive interpretation in the product evaluation process; Understanding the consumer's decision and intention process.
Cognitive product design to innovation	Structure to explain the dynamics of cognition and emotion in the perceived value of symbolic and aesthetic properties of the product (Rindova and Petkova, 2007). Measure of satisfaction with innovative design and the visceral, behavioral, and reflective attributes of a car steering system (Li et al., 2014).	Create cognitive and emotional psychological effects through a product's form; Improve the perception of value; Facilitate the understanding and comprehension of the product through aesthetics; Make consumption more meaningful and enjoyable through aesthetic form.
Affective-cognitive experience product design	Cognitive and affective measures of user experience, their decision-making process, and understanding of integration of the cognitive and affective systems (Zhou et al., 2013). Cognitive and affective decision measures to understand how subjective experience and affective prediction influence the behavior of choice under uncertainty (Jiao et al., 2017).	Personalizing mass products using the implicit data available on the web; Improve user experience through cognitive-affective product design; Evaluate the product in real time from physiological data; Integrate cognitive-affective systems in assessments; Develop an analytical model of the consumer's cognitive-affective decision; Reveal trends in cognitive and affective biases in consumer decision making.

The main challenges in applications define the current state of cognitive and affective approaches to product design.

### State of the Art of Applying Consumer's Cognitive and Affective Needs in Product Design

**Figure 5:**
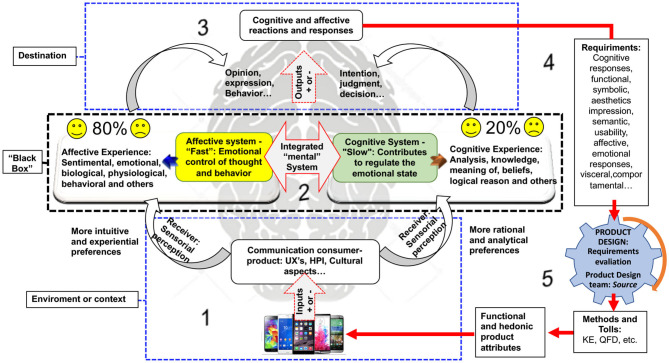
State of the art of applying consumer's cognitive and affective needs in product design.

For Wrigley (2013), 80% of an individual's life is consumed by their emotions, while the other 20% is controlled by their intellect. Emotions directly influence a variety of cognitive responses, and research on emotional effects on consumer choice is an important field which is little studied by designers and developers (Hirschman and Stern, 1999). At this point the state of the art is structured, where the status of applications and common challenges are summarized and presented in five stages that integrate a cognitive and affective product design cycle as illustrated in Figure 5.

In the first *stage* (Figure 5—Detail 1), most applications' cognitive and affective needs in product design take place in the context of experience between the product and the consumer (Kumar and Garg, 2010; Zhou et al., 2013; Jiao et al., 2017; Hsu et al., 2018). Product input attributes can be perceived sensibly as “positive” or “negative.” In the initial communication stage, rational preferences, analytical, intuitive, and experimental (beliefs, memories, and others) should be encouraged by the product attributes that can be functional, cognitive, hedonic, or affective (Blackler et al., 2010; Wrigley, 2013).

In the second *stage* (Figure 5—Detail 2), the functional and hedonic attributes of the product are processed by the “cognitive and affective systems” of the consumer on a single integrated mental process (Khalid and Helander, 2004, 2006; Khalid, 2006). This is understood by most researchers as a “black box” complex and a difficult to understand assessment (Zhou et al., 2013; Diego-Mas and Alcaide-Marzal, 2016; Jiao et al., 2017). At this point, what happens is the subjective product experience, in which the bias is not known. However, the systems link different weights and measures which account for the decision-making process (Kahneman and Tversky, 1979; Jiao et al., 2017). The emotional system is higher (80%) compared to the cognitive system (20%) (Wrigley, 2013). The result of subjective product experience can be expressed in intentions (Giese et al., 2014; Yang et al., 2016; Wang et al., 2018), quality judgments (Page and Herr, 2002; Hsu, 2017), decisions (Dogu and Albayrak, 2018), opinions, and attitudes. The expressions shown in the *third step* (Figure 5—Detail 3) represent the reactions and cognitive and affective responses (positive and negative outputs) and are intended by the design team and product engineering to result in response requirements of subjective product experience (Figure 5—Detail 4).

The outputs are understood as necessary entry requirements for the *fourth stage* (Figure 5—Detail 4). The requirement can be a cognitive response, functional (Khalid and Helander, 2004; Rindova and Petkova, 2007; Seva et al., 2011; Homburg et al., 2015), aesthetic (Artacho-Ramírez et al., 2008; Kumar and Garg, 2010; Carbon and Jakesch, 2013; Greggianin et al., 2018; Wiecek et al., 2019), symbolic semantics (Demirbilek and Sener, 2003; Crilly et al., 2004; Rindova and Petkova, 2007; Artacho-Ramírez et al., 2008; Setchi and Asikhia, 2019), usability (Seva et al., 2011; Li and Gunal, 2012), emotional (Demirbilek and Sener, 2003; Kumar and Garg, 2010), visceral (Wrigley, 2013; Aftab and Rusli, 2017), and others. At this time, these requirements must be evaluated and translated by engineering and product design teams (Li et al., 2014).

Finally, in the fifth *step* (Figure 5—Detail 5), the product design teams must evaluate the consumer response requirements through models, methods, and tools for evaluation and translation such as kansei engineering, quality function deployment, among others (Huang et al., 2012; Li et al., 2014; Yuen, 2014; Shen and Wang, 2016).

Figure 5 provides designers with reasonable guidelines for comprehensively capturing, evaluating, and translating customer requirements. In this sense, it seeks to convert subjective consumer information into product design demands and processes and select the technical requirements for functional, usability, hedonic, and holistic improvements in the product. The product is then designed and developed in a targeted way for the cognitive and affective subjective satisfaction of consumers, helping designers in search of “cognitive” and “affective” solutions for the product. At this point, the product design application cycle, usually oriented toward the consumer, starts again in a cyclical manner.

### Advances in Neuroscience

Neuroscience addresses the importance of multidisciplinary knowledge in order to understand the opinions and consumer responses to cognitive and affective product design. Can a model potentially influence decision processes including price, choice strategy, context, experience, and memory; and also provide new insights into individual differences in consumer behavior and brand preferences? The fundamental question, still little evidenced, is how to apply these neuroscience advances in product design, making the product more accessible, more comfortable, and more enjoyable to use and consume.

According to Maturana and Varela (1987), if the goal is to understand any human activity, then it is necessary to consider the emotion that defines the field of action in which this activity takes place and in the process, learn to observe what actions the emotion you want. Intentions start from the subjective, emotional, and affective internal processes that are expressed. It is essential to understand in-depth the phenomenon of subjective experience. Wrigley (2011, 2013) attested that the response elements of “emotional cognition” are not presented as objective qualities of a product. However, these elements are a cognitive interpretation of the qualities of an object, driven both by the perception of real stimuli and by facts evoked by the consumer's memory and emotion. It affects the facial muscles and the musculoskeletal structure, also the visceral and internal environment of the body as well as the neurochemical responses in the brain and are part of how emotions modify the internal state of the body. Damasio (2001) described it similarly as in their exploration noted that the instinctive, visceral, and immediate response to sensory information strongly influenced the secondary information acquired when cognitive-behavioral interaction and reflection occurred later. There is a hierarchy of internal processes in operation, for although the affection and cognition are, to some extent, different neuroanatomically systems, they are deeply interconnected, influencing each other (Ashby et al., 1999; Crilly et al., 2004; Norman, 2004).

Traditional assessment methods rarely present a complete understanding of user's cognitive and affective experience evoked by the product, which plays a decisive role in intention and purchase decision. Regarding product design, Ding et al. (2016) present a method of accurate measurement of user perception during product experience. The results of the application revealed a neural mechanism in the initial stage of the consumer experience, allowing for an accurate analysis of the time course of neural events when the behavioral intention is forming. Such advances can provide a basis for discovering the cognition and decision process when users perceive product design, and even provide help for the designer to hold the user's attention. Modica et al. (2018) stated that evaluation of a product considers the simultaneous cerebral and emotional evaluation of different qualities of the product, all belonging to the product experience. They investigate reactions by electroencephalographic (EEG) of the influence of brand, familiarity, and hedonic value, and results show more significant mental effort during an interaction with foreign products which demonstrates the importance of the perceived ease of a product. Also, concerning the use of neurophysiological and traditional measures to evaluate the responses of the participants through an EEG index (EEG), Martinez-Levy et al. (2017) pointed out that the change in EEG frontal cortical asymmetry is related to the general appraisal perceived during an observation of a charity campaign focusing on gender differences. Results show higher values for women than men for neurophysiological indices. Therefore, the declared taste of women is statistically significantly higher than the declared taste of men. Results suggest the presence of gender differences in cognitive and emotional responses to charity ads with emotional appeal. By providing a new way of establishing mappings between cognitive processes and traditional marketing data, Venkatraman et al. (2012) point out that a better understanding of neural decision-making mechanisms will increase the ability of marketers to market their products more effectively.

Neuroscience applied to the product market and psychology has brought significant advances in the last 20 years to the understanding that cognitive and emotional aspects generate greater consumer involvement. The objective is to further reduce the gap between product and consumer. New insights into individual differences in consumption behavior and specific preferences are presented. It also contributes to advances in the area of cognitive and affective product design, however still firmly positioned in areas of marketing and psychology.

### Research Gaps in Literature

Cognitive design approaches have been proven to be a less discussed topic by the leading authors in the field, while affective/emotional design approaches are the most applied. The reason for this is that cognitive design is more associated with the product functionality and usability, the focus on ergonomics and systems engineering, in addition to interfaces and systems aimed at product use and not necessarily at consumption. Therefore, cognitive design approaches are slightly different from affective/emotional design approaches. These are more oriented to the design, form, and impact of the product attributes on the consumer's feelings and emotions. This way, they are mainly directed to product pleasure and pleasantness.

The areas of product design, engineering, and ergonomics are mixed in applications that focused on product design and on how functional and “cognitive” attributes, as well as hedonic and “affective” ones, affects the consumer's reactions and responses. The results of the SLR indicate that researchers paid predominant attention to areas of how cognitive and affective aspects can be applied in product design, and concentrated at the beginning of the PD and NPD cycle, that is, when evaluating and translating the consumer's reactions and responses when using or consuming the product.

In short, cognitive approaches are more up-to-date and associated with technology, and are therefore aimed at the ease and friendliness of the product. In contrast, affective approaches are older and aimed at quality of life, satisfaction, pleasure, and the pleasantness of the product. Due to the complexity of understanding the affective and emotional subjectivity of the consumer, and in how to translate these requirements into product attributes, these approaches seem to lose their preference in the areas of design and engineering for cognitive applications.

Some approaches identify the importance of an integrated application framework that considers all consumer's cognitive and affective aspects. However, they do not deepen the study on the intrinsic phenomenon of the subjective experience resulting from cognitive and affective systems, inherent to “mental” processes, which opens an essential gap for research (Khalid and Helander, 2006; Zhou et al., 2013; Jiao et al., 2017). The trends point to the need to decipher the complexity of the “black box” of human subjectivity and, thus, influence consumer behavior.

### Future Directions and Research

The main trends in the research field refer to: (i) studies on the consumer's sensory, cognitive, and affective perception (Wrigley, 2013) concerning the product's functional and hedonic attributes and characteristics (Khalid and Helander, 2004, 2006); (ii) studies on the consumer's subjective cognitive and affective experience about the product (Jiao et al., 2017); and (iii) studies on capturing, measuring, and translating consumers' cognitive and affective responses and opinions (Crilly et al., 2004; Hsu et al., 2018).

Therefore, from the individual approaches in each article, it is possible to observe the researchers' acceptance that the consumer's subjective experience begins through sensory and cognitive perception. When it is perceiving and processing the inputs from the product (functional and hedonic characteristics and attributes, for example); then, by the psychological processing of the cognitive (slow) and affective (fast) systems (Kahneman and Tversky, 1979; Kahneman, 2011) it brings memories of previous experiences, beliefs, images, and emotions; and finally ends with responses and opinions, with cognitive and affective elements (Crilly et al., 2004; Khalid and Helander, 2004; Kumar Ranganathan et al., 2013; Zhou et al., 2013; Jiao et al., 2017; Hsu et al., 2018).

Among the topics and questions to be considered in future research, we suggest: what are the psychological relationships between the cognitive and affective needs of the consumer in the use or consumption of products? What characteristics and attributes of the product have a positive cognitive and affective impact on the consumer? Through product design and new products, is it possible to produce pleasure and happiness in the consumer's mind? Can an inclusive product design facilitate use in populations with increasing cognitive difficulties? Can we develop better predictive models to anticipate the consumer's intention and decision when choosing products?

## Conclusions

The aim of this study was to investigate the cognitive and affective needs of the consumer applied to product design through a systematic literature review of the literature published in the last 20 years. In this regard, this article selected the main research carried out in the field of cognitive and affective product design and identified the main approaches, challenges, and trends in applications.

Among the different approaches analyzed, there were research fields that seek to understand the consumer's behavior, emotions, affections, and reflections on the product. Cognitive and affective product design follows this path and seeks to narrow the space between the product and the consuming public. However, cognitive approaches were less discussed than affective ones. The possibility of cognitive design was more associated with the product's functionality and usability, interfaces, and systems—usually the focus of ergonomics and systems engineering—and not necessarily consumption, which was clearly the focus of affective design and marketing. The areas of product design, engineering, and ergonomics mix with applications that focus their efforts on how functional and “more cognitive” attributes and characteristics, as well as hedonic and “more affective” attributes and characteristics, affect the consumer's reactions and responses. They indicate that applications that are both cognitive and affective open an important path for future research on consumer-oriented product design. The goal is always to improve the interaction or the consumption experience by facilitating the information flow, thus improving communication between consumer and product, positively affecting them.

As a synthesis for the approaches, it is possible to conclude that applications in “usability,” “cognitive ergonomics,” and “cognitive engineering” are more current than applications in “affective design,” “kansei engineering,” and “emotional design.” All the applications analyzed are interconnected to product design and indicate that cognitive approaches are more focused on product usability and functionality, while the affective/emotional approaches are more focused on pleasure and consumption. These characteristics are important for the consumer study, as it applies to product design that is still in the conceptualization phase, exactly where the approaches are oriented to the evaluation and translation of the consumer's subjective responses.

In short, cognitive approaches are more up-to-date and associated with technology, therefore aimed at the ease and friendliness of the product. While affective approaches are older and aimed at quality of life, satisfaction, pleasure, and the pleasantness of the product. This review indicates that this shift in focus from the affective to the cognitive is due to the complexity of understanding the affective and emotional subjectivity of the consumer and how to translate these requirements into product attributes, these approaches seem to lose their preference in the areas of design and engineering for more rational and logical cognitive applications, making them therefore more statistically verifiable.

Finally, this study recommends that, in future research, the objective should be to create analytical methods and tools (Zhou et al., 2013; Jiao et al., 2017), with multidisciplinary approaches (Jiang et al., 2015a; Chan et al., 2018) from different areas of consumer study such as engineering and design (Jiang et al., 2015b; Shen and Wang, 2016), marketing (Seva et al., 2007; Bloch, 2011; Mu, 2015), neuroscience, and cognitive sciences (Damasio and Adolphs, 2001; Turner and Laird, 2012), while seeking to evaluate and translate the consumer's subjective experience into product elements and attributes. The objective is to improve the relationship between the consumer and the product, making it lighter and with a better information flow.

We conclude that it is necessary that approaches to cognitive and affective product design be incorporated into research about the consumer, so that no need, be it more functional and cognitive or more pleasurable and affective, is left unattended. Thus, it will be possible to bring the consumer closer to the product, meeting their subjective needs, and to open the “black box” of subjective experience that only the consumer themselves have access to. In this way, it will become possible to meet the cognitive and affective needs of the consumer and produce happiness in their mind, something essentially subjective and understood as difficult to evaluate and translate. The cognitive design must be mixed with affective design, as in a high-tech world, the product's facilities and usability are producing affective pleasure in the consumer through the economy of cognitive effort.

### Research Limitations

There are limitations to this research. The next step in the research should focus on finding new methods and models for evaluating and translating the cognitive and affective product experience, with combined psychological and physiological measures, according to what Zhou et al. (2013) previously suggested. The present study only focused on two dimensions of cognitive and affective product design: the cognitive and affective/emotional attributes and characteristics. However, the authors suggest that the symbolic dimension presents significant differences when compared to the cognitive and affective aspects, following the studies carried out by Bloch (2011), Kumar Ranganathan et al. (2013), and Homburg et al. (2015).

The path of opportunities lies in multidisciplinary approaches that consider neuroscience and cognitive sciences, together with cognitive and affective product design, as well as their current understandings on the themes highlighted in this research. The deepening of these questions is a limitation of this research. The authors understand the need to continue research on analytical methods and models capable of improving the understanding of the affective and cognitive decision-making process regarding product design. New analytical tools must be oriented toward the consumer and their subjective experiences. These can translate opinions and responses from the “black box” or the subjective experience of the product.

## Data Availability Statement

The original contributions presented in the study are included in the article/supplementary material, further inquiries can be directed to the corresponding author/s.

## Author Contributions

All authors listed have made a substantial, direct and intellectual contribution to the work, and approved it for publication.

## Conflict of Interest

The authors declare that the research was conducted in the absence of any commercial or financial relationships that could be construed as a potential conflict of interest.
